# C-factor: a summary measure for systemic arterial calcifications

**DOI:** 10.1186/s12872-021-02126-y

**Published:** 2021-06-29

**Authors:** Lieke M. Kuiper, M. Kamran Ikram, Maryam Kavousi, Meike W. Vernooij, M. Arfan Ikram, Daniel Bos

**Affiliations:** 1 Departments of Epidemiology, Erasmus MC, P.O. Box 2040, 3000 CA Rotterdam, The Netherlands; 2 Department of Neurology, Erasmus MC, Rotterdam, The Netherlands; 3 Department of Radiology and Nuclear Medicine, Erasmus MC, Rotterdam, The Netherlands

**Keywords:** Arterial calcification, Arteriosclerosis, Biomarker, Epidemiology, Imaging

## Abstract

**Background:**

Arterial calcification, the hallmark of arteriosclerosis, has a widespread distribution in the human body with only moderate correlation among sites. Hitherto, a single measure capturing the systemic burden of arterial calcification was lacking. In this paper, we propose the C-factor as an overall measure of calcification burden.

**Methods:**

To quantify calcification in the coronary arteries, aortic arch, extra- and intracranial carotid arteries, and vertebrobasilar arteries, 2384 Rotterdam Study participants underwent cardiac and extra-cardiac non-enhanced CT. We performed principal component analyses on the calcification volumes of all twenty-six possible combinations of these vessel beds. Each analysis’ first principal component represents the C-factor. Subsequently, we determined the correlation between the C-factor derived from all vessel beds and the other C-factors with intraclass correlation coefficient (ICC) analyses. Finally, we examined the association of the C-factor and calcification in the separate vessel beds with cardiovascular, non-cardiovascular, and overall mortality using Cox–regression analyses.

**Results:**

The ICCs ranged from 0.80 to 0.99. Larger calcification volumes and a higher C-factor were all individually associated with higher risk of cardiovascular, non-cardiovascular, and overall mortality. When included simultaneously in a model, the C-factor was still associated with all three mortality types (adjusted hazard ratio per standard deviation increase (HR) > 1.52), whereas associations of the separate vessel beds with mortality attenuated substantially (HR < 1.26).

**Conclusions:**

The C-factor summarizes the systemic component of arterial calcification on an individual level and appears robust among different combinations of vessel beds. Importantly, when mutually adjusted, the C-factor retains its strength of association with mortality while the site-specific associations attenuate.

**Supplementary Information:**

The online version contains supplementary material available at 10.1186/s12872-021-02126-y.

## Background

Arteriosclerosis is the single most important cause of coronary heart disease and stroke [1]. Increasingly, computed tomography (CT) has become a key asset to study arteriosclerosis given its ability to detect calcification, which is one of the most prominent hallmarks of the disease [2].

In the past decade, the location of arterial calcification has become an important topic of interest [3, 4]. It has emerged that the burden of calcification within an individual may vary substantially across vessel beds. From a clinical perspective, these vessel-specific differences harbor unique information with regard to the risk of subsequent clinical sequela, e.g., myocardial infarction in the case of the coronary arteries or stroke in the case of the carotid arteries [5, 6]. In addition to the vessel-specific burden of arterial calcification, it is important to realize that arterial calcification may develop anywhere in the arterial system. In this light, the use of full-body scans has even been suggested to obtain information on the systemic burden of arterial calcification [7]. As there is a large variety in size of the different vessel beds, a calcification sum score or mean will be mostly dependent on the amount of calcification in the larger vessel beds, such as the aorta. Hence, to take these differences into account, more advanced data reduction techniques are required to compose a single summary measure for the systemic component of arterial calcification.

Advantages of such a single measure of arterial calcification can be expected on four levels. First, this measure will provide an estimate of the overall burden of systemic arterial calcification within one individual, using a limited number of vessel beds. Second, it could facilitate a more accurate indication of the health status of a person beyond cardiovascular risk as information from different parts of the body are included in the measure. Third, it can be used to monitor the effects of systemic medical treatment for cardiovascular risk reduction in trials or clinical practice. Fourth, it will provide the opportunity to compare systemic arterial calcification across individuals that do not necessarily have imaging of the same vessel beds available.

Importantly, substantial advantages of single such summary measures have been demonstrated in other fields of medicine. The cognitive neuroscience field uses the g-factor as a domain-independent summary measure of global cognition. The g-factor is stable among different test batteries [8, 9]. The frailty index is used in older populations to summarize the state of vulnerability to adverse health outcomes [10]. Against this background, in this study, we propose a novel summary measure for arterial calcification, the “C-factor” and determine its association with all-cause and cause-specific mortality.

## Methods

### Study population

This study is embedded in the Rotterdam Study, a population-based cohort study based in Ommoord, a suburb of Rotterdam, that started in 1990 and includes over 15,000 participants [11]. All participants were examined at study entry and return for re-examination every 3 to 5 years. Every participant who came for re-examination between 2003 and 2006 (n = 3229) was asked to undergo non-contrast multidetector computed tomography (CT) to assess arterial calcification; 2524 of them (response rate of 78%) underwent the CT [4, 6, 12]. For the current study, we restricted our sample to the 2384 participants with complete data on calcification in the coronary arteries, the aortic arch, the extracranial carotid arteries, intracranial carotid arteries, and the intracranial vertebrobasilar arteries.

### Assessment of arterial calcification

Non-contrast CT-images were obtained using a 16-slice or 64-slice multidetector CT-scanner (Somatom Sensation 16 or 64; Siemens, Forchheim, Germany). Participants underwent an ECG-gating cardiac scan and an extra-cardiac scan that reached from the aortic root to the intracranial vasculature (1 cm above the sella turcica). The estimated radiation dose was up to 2.1 mSv during the cardiac scan. The estimated dose was 2.8 mSv during the extra-cardiac scan. The cardiac scans of a small proportion of the participants with a heart rhythm disorder required a radiation dosage up to 4.1 mSv. Each participant was scanned once [3, 12]. Using these scans, we visualized the following vessels: coronary arteries, aortic arch, extracranial carotid arteries, intracranial carotid arteries, and vertebrobasilar arteries. We set the threshold of calcification on 130 Hounsfield units [13]. We quantified the volume of calcification (in mm^3^) as the volume above the threshold in the coronary arteries (CAC), the aortic arch (AAC), and the extracranial carotid arteries (ECAC) automatically, using specialized software (Syngo Calcium Scoring, Siemens, Forcheim, Germany). Due to the close relation of the bony skull base and the calcium in the wall of the intracranial carotid arteries (ICAC) and vertebrobasilar arteries (VBAC), these vessel beds could not be quantified automatedly. We, therefore, quantified calcification in these vessel beds semi-automatically. A more detailed description can be found elsewhere [14–16].

### Assessment of mortality

We obtained information on the vital status of the participants through the mortality registry of the municipality and the digitally connected medical records of the general practitioners working in the study area on a bimonthly basis [17, 18].

After notification of the death of a participant, information on the cause and circumstances of death were requested from the medical records of the general practitioners or the nursing home physicians. Research physicians categorized the cause of death based on ICD-10 criteria [17–19]. In this study, cardiovascular mortality was defined as arteriosclerosis-related cardiovascular mortality and hence seen as mortality coded with the following ICD-10 codes: I21, I25, I26, I33, I35, I42, I46, I50, I61-I64, I69, I70, I71, I73, I74, and I99. When a patient died during the study time, but the cause of death was not coded with one of the ICD-10 codes above, this was defined as non-cardiovascular mortality. Follow-up for mortality was complete until the 1st of January 2015.

### Assessment of covariates

We used interviews, physical examinations, and blood sampling to gather information on current smoking, obesity, hypertension, hypercholesterolemia, low high-density lipoprotein cholesterol, and diabetes mellitus [20–22]. We defined prevalent cardiovascular disease as having had a stroke or myocardial infarct or having undergone a coronary artery bypass graft or percutaneous coronary intervention prior to the CT-scan.

### Statistical analysis

To obtain the C-factor, we performed a principal component analysis without varimax rotation (PCA), a data reduction method that simplifies the complexity in high-dimensional data. This data reduction is accomplished by geometrically projecting the data on lower dimensions, named principal components, that are uncorrelated. We performed the PCA based on the correlation matrices of the calcification in the different vessel beds because this standardizes the different variables included in the analysis. Standardization is a crucial step in our analysis since the vessel beds vary in size; otherwise, the larger vessel beds would drive the analysis. Loadings are the linear combination weights or coefficients of the PCA. The geometrical projection makes the loading on an arbitrary basis positive or negative. For the same analysis, the assigned sign can differ among statistical software used. Therefore, we fixed the loadings to ensure that the first element of each loading is non-negative; this makes the outcomes robust among statistical software. The first principal component entails, by definition, the most variance and, thus, most information [23, 24]. Therefore, the first principal component that resulted from our analysis was coined the C-factor. We performed the PCA in all participants, and men and women separately.

As a quality control procedure, we examined the scree plot of eigenvalues of the different principal components and the total variance explained by the C-factor. Ideally, one prefers to select only principal components with an eigenvalue ≥ 1.0, because this indicates that the principal component explains at least as much variance as a single variable put in the PCA. Furthermore, every principal component accounts for a certain amount of the variance between the variables. We explored the variance explained by the C-factor to get insight into the amount of information it entails.

Thereafter, we performed the PCAs and quality control procedure for all the different combinations of four, three, and two vessel beds (25 combinations). Afterward, we determined the intraclass correlation between the C-factor based on calcification volumes of all five vessel beds, i.e., the overall C-factor, and the C-factors based on the different combinations of calcification volumes of two, three or four vessel beds, to examine the robustness of the C-factor. As described previously, calcification volumes in the separate vessel beds have correlation coefficients ranging from 0.24 to 0.60 [4, 12, 15]. Hence, the overall C-factor should have an intraclass correlation coefficient of at least 0.60 with the other C-factors to outperform the correlation between all the separate vessel beds. Therefore, we set a threshold of an intraclass correlation of 0.60 to claim that this parameter captures the systemic component of arterial calcification [4, 12, 15]. We made histograms of the calcification volumes in the separate vessel beds and the overall C-factor. We plotted the calcification volumes and C-factor of five case examples in those histograms to give insight in the composition of the C-factor.

We transformed the overall C-factor and the calcification volumes in the separate vessel beds because of their skewed distribution [25, 26]. In the case of the overall C-factor we performed a fourth-root transformation. Prior to this transformation, we translated the C-factor to a minimum of 0 to deal with the majority of negative outcomes, we translated the C-factor to a minimum of 0 ($\sqrt[4]{C-factor}$ (after transformation to solely positive values)). As a fourth-root transformation is an uncommon transformation, we performed a sensitivity analysis with the more common 10th-root-transformation. The results were similar for the 10th-root-transformation of the overall C-factor (data not shown), but the histogram indicated that the 4th-root-transformation fitted the data best. In the separate vessel beds, we performed a natural log-transformation after adding 1.0 mm^3^, to deal with participant without calcification in the vessel beds, to the calcification volumes (Ln[calcification volume + 1.0 mm^3^]). We imputed missing values (with a maximum proportion of 6.1%) on cardiovascular risk factors using fivefold multiple imputation, based on calcification of the different vessel beds, age, sex and other cardiovascular risk factors [27].

Next, using Cox Proportional hazards regression models, we determined the association of the overall C-factor (per standard deviation increase) with the broad outcome of mortality, which was measured as cardiovascular mortality, non-cardiovascular mortality, and overall mortality. We compared the association between the C-factor and the broad outcome of mortality with associations of calcification in the separate vessel beds (per standard deviation increase) with these endpoints. We used three different models for the analyses. Model 1—adjusted for age and sex. Model 2—adjusted for traditional cardiovascular risk factors. Model 3—adjusted for age, sex, the overall C-factor, and calcification in all vessel beds. We performed a sensitivity analysis, to check if our results were not driven by participants with prevalent cardiovascular disease, on the association between the C-factor and mortality. In the sensitivity analysis, we excluded participants with prevalent cardiovascular disease and participants of whom information on prevalent cardiovascular diseases was not available. We defined prevalent cardiovascular diseases as having had a stroke or myocardial infarct or having undergone a coronary artery bypass grafting or percutaneous coronary intervention prior to the CT-scan. All statistical analyses were performed in R version 3.5.3 (R Foundation for Statistical Computing, Vienna, Austria), and we used the psych, mice, ggplot2, and survival packages to perform our analyses.

## Results

### Characteristics of the study population

The mean age of the 2384 participants was 69.6 years, and 1254 (52.6%) were women. The median volume of CAC was 52.1 mm^3^; AAC 258.3 mm^3^; ECAC 22.5 mm^3^; ICAC 43.0 mm^3^; and VBAC 0.0 mm^3^ (Table 1).

**Table 1:** Characteristics of the study population

Sample size	2384
Women	1254 (52.6)
Age, years	69.6 ± 6.8
Currently smoking	380 (15.9)
Obesity	573 (24.0)
Diabetes mellitus	314 (13.2)
Hypertension	1766 (74.1)
Hypercholesterolemia	1172 (49.2)
Low high-density lipoprotein cholesterol	264 (11.1)
Prevalent cardiovascular disease*	238 (10.0)
Presence of CAC	1957 (82.1)
CAC-volume, mm^3^	52.1 [2.0–273.4]
Presence of AAC	2210 (92.7)
AAC-volume, mm^3^	258.3 [44.9–841.1]
Presence of ECAC	1745 (73.2)
ECAC-volume, mm^3^	22.5 [0.0–114.6]
Presence of ICAC	1951 (81.8)
ICAC-volume, mm^3^	43.0 [7.0–141.3]
Presence of VBAC	486 (20.4)
VBAC-volume, mm^3^	0.0 [0.0–0.0]

Presented values are mean ± standard deviation for age, median [Q1–Q3] for the volumes of calcification of vessel beds and n (%) for dichotomous variables

AAC, aortic arch calcification; CAC, coronary artery calcification; ECAC, extracranial carotid artery calcification; ICAC, intracranial carotid artery calcification; and VBAC, vertebrobasilar artery calcification

^*^Information on prevalent cardiovascular disease was missing in 11 participants

### Composing the overall C-factor

**Fig. 1:**
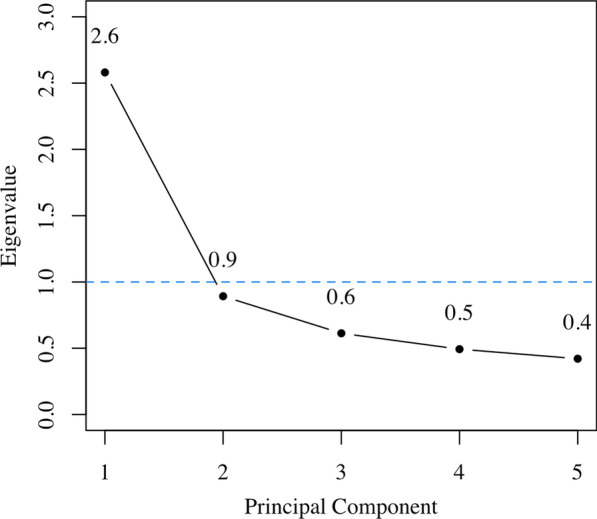
Scree plot of the C-factor including calcification volumes of all five vessel beds. The scree plot shows the eigenvalues plotted over the principal components. The dotted blue line represents the threshold of an eigenvalue of 1.0, above which a principal component explains at least as much variance as a single variable put in the principal component analysis. Only the first principal component, the C-factor, is above this threshold

**Fig. 2:**
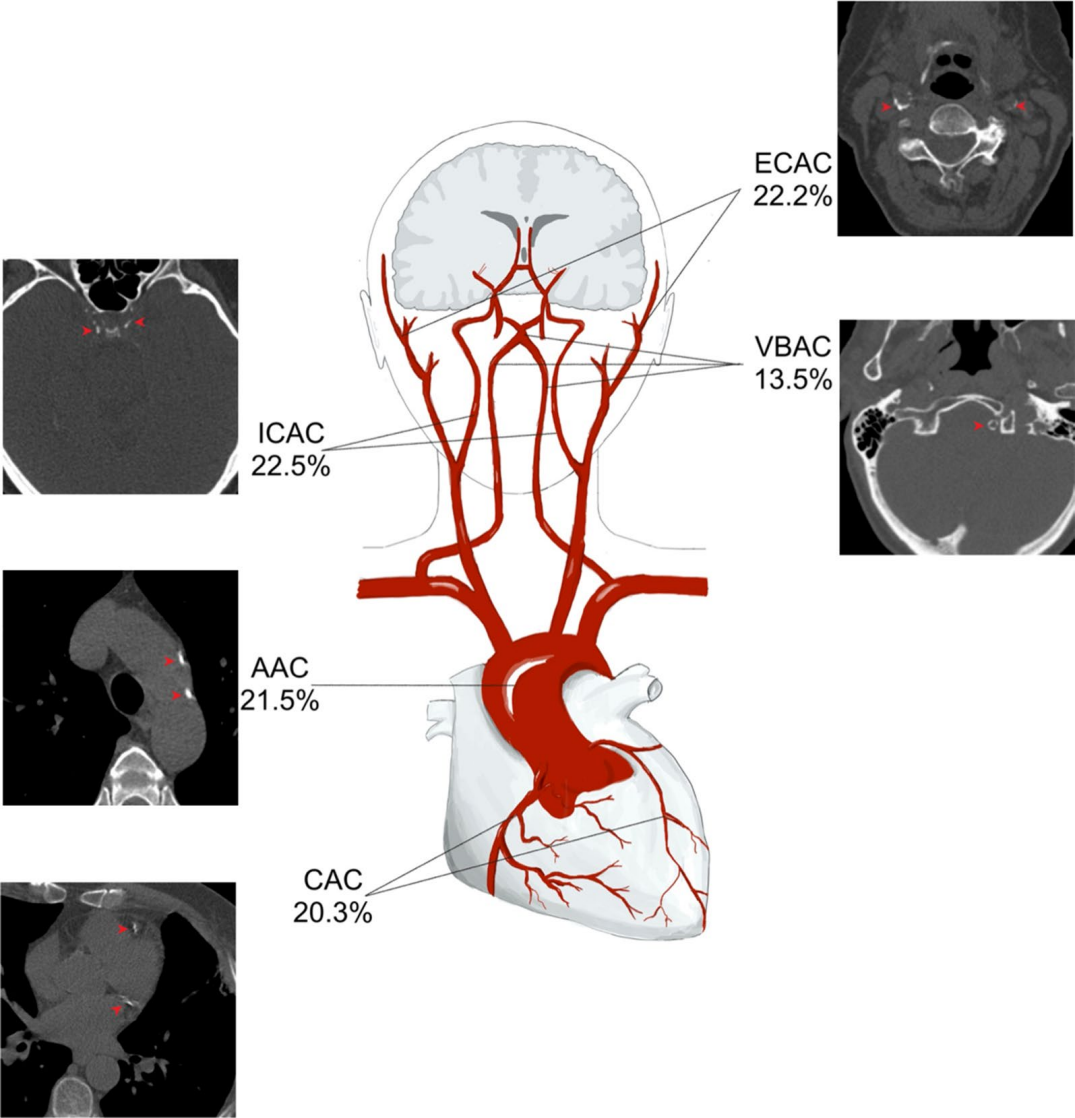
Contribution of calcification in the different vessel beds, in percentage, to the overall C-factor. The CT-images show examples of calcification in the different vessel beds. Calcification is indicated with a red arrow. AAC indicates aortic arch calcification; CAC, coronary artery calcification; ECAC, extracranial carotid artery calcification; ICAC, intracranial carotid artery calcification; and VBAC, vertebrobasilar artery calcification

Preliminary research suggested that the composition of the C-factor did not differ between the sexes (Additional file 1: Table S1). Hence the presented results are based on principal component analyses including all participants. Figure 1 displays the scree plot from the overall C-factor. The second eigenvalue is under the threshold of 1.0. The total explained variance of the C-factor was 52% (Table 2). The standardized score of ICAC contributed most to the C-factor, and the standardized score of VBAC the lowest, as is shown in Fig. 2.

**Table 2:** Combination-specific C-factor, total explained variance and its intraclass correlation with the overall C-factor

Combination-specific C-factor based on	Total explained variance by the combination specific C-factor	Intraclass Correlation with overall C-factor
CAC, AAC, ECAC, ICAC, VBAC	0.52	1.00
CAC, AAC, ECAC, ICAC	0.61	0.99
CAC, AAC, ECAC, VBAC	0.52	0.98
CAC, ECAC, ICAC, VBAC	0.53	0.98
CAC, AAC, ICAC, VBAC	0.52	0.98
AAC, ECAC, ICAC VBAC	0.55	0.98
CAC, AAC, ECAC	0.65	0.97
CAC, AAC, ICAC	0.63	0.97
CAC, AAC, VBAC	0.53	0.94
CAC, ECAC, ICAC	0.65	0.97
CAC, ECAC, VBAC	0.53	0.95
CAC, ICAC, VBAC	0.57	0.93
AAC, ECAC, ICAC	0.67	0.97
AAC, ECAC, VBAC	0.56	0.95
AAC, ICAC, VBAC	0.57	0.95
ECAC, ICAC, VBAC	0.57	0.95
CAC, AAC	0.70	0.91
CAC, ECAC	0.72	0.92
CAC, ICAC	0.73	0.92
CAC, VBAC	0.61	0.80
AAC, ECAC	0.78	0.92
AAC, ICAC	0.73	0.93
AAC, VBAC	0.61	0.83
ECAC, ICAC	0.76	0.93
ECAC, VBAC	0.59	0.85
ICAC, VBAC	0.68	0.82

AAC, aortic arch calcification; CAC, coronary artery calcification; ECAC, extracranial carotid artery calcification; ICAC, intracranial carotid artery calcification; and VBAC, vertebrobasilar artery calcification

**Fig. 3:**
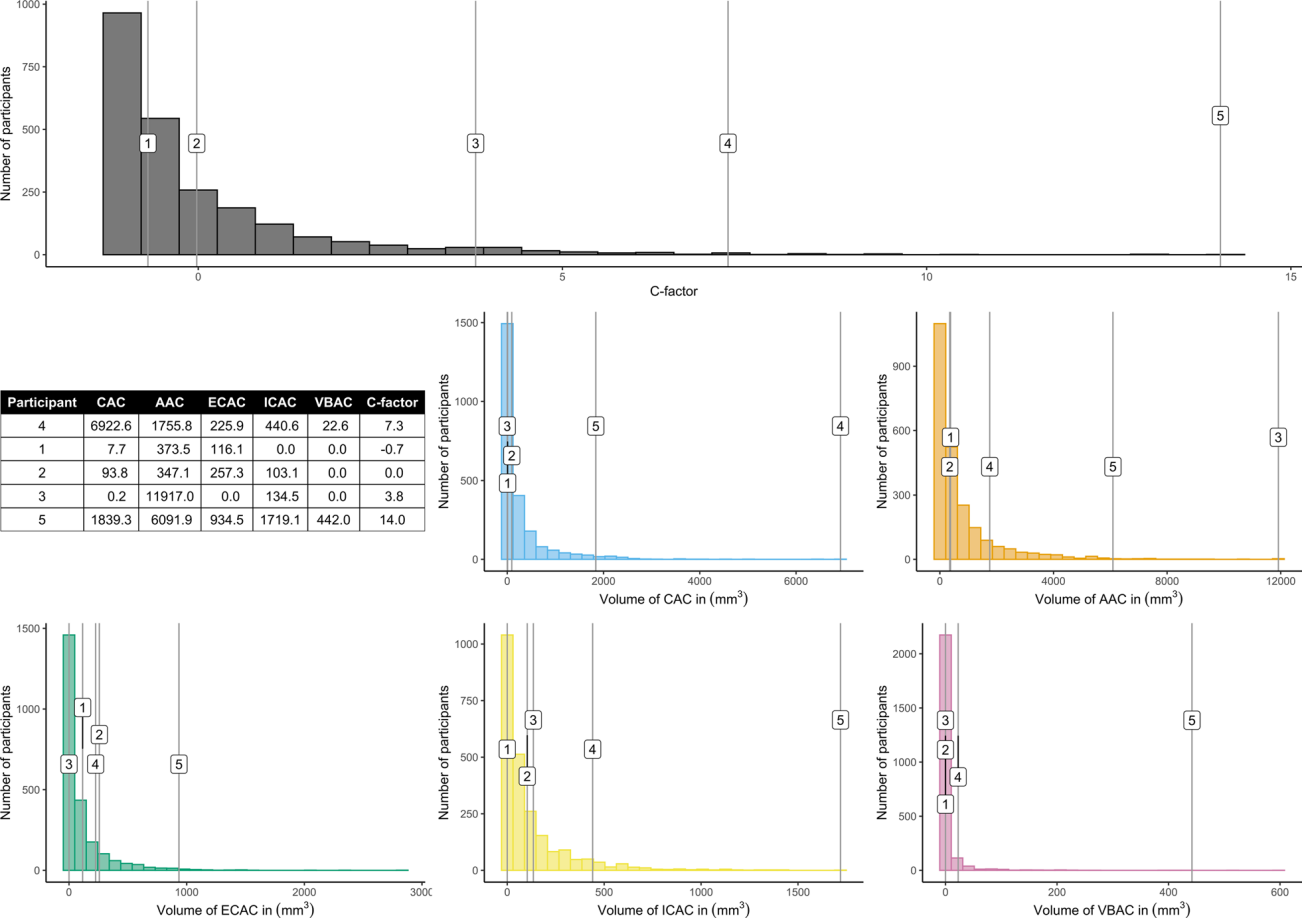
Composition of the overall C-factor for five example cases. The upper histogram shows the frequency distribution of the overall C-factor. The table shows the calcification information on the five vessel beds included in the overall C-factor and the assigned C-factor of the five example cases. The other histograms show the frequency distribution of the different vessel beds. The calcification volumes in the table are in mm^3^. The numbers that are indicated in the histograms represent the five participants. To clarify, number 1 indicates participant one in all six histograms. AAC indicates aortic arch calcification; CAC, coronary artery calcification; ECAC, extracranial carotid artery calcification; ICAC, intracranial carotid artery calcification; and VBAC, vertebrobasilar artery calcification

Figure 3 shows five representative cases of the calculation of the C-factor in our population. Case 1 shows a participant with an ECAC volume in the third quartile but a moderate to low calcification in the other vessel beds. This results the lowest C-factor of the five. Case 2 had moderate calcification in all five vessel beds, which resulted in a higher C-factor than Case 1. Case 3 had an extremely large volume of AAC, a high ICAC volume, and moderate to low volume of calcification in the other vessel beds. Case 4 had the highest volume of AAC but not the highest C-factor as Case 5 had higher calcification volumes in the other vessel beds.

### The systemic component of the arterial calcification

All the C-factors derived from calcification volumes of four, three, and two separate vessel beds had an intraclass correlation coefficient with the overall C-factor above the threshold of 0.60 (Table 2). The lowest intraclass correlation was found between the overall C-factor and the C-factor consisting of CAC and VBAC. The C-factor consisting of CAC, AAC, ECAC, ICAC had the highest correlation with the overall C-factor. The descriptive statistics of all combination-specific C-factors can be found in Additional file 1: Table S2.

### Cardiovascular mortality, non-cardiovascular mortality, and overall mortality

A total of 456 participants died during 21,693.5 person-years of follow-up (median follow-up time 9.6 years), of which 121 died as a consequence of a cardiovascular cause. A higher C-factor or larger calcification volume in any vessel bed was associated with a higher risk of cardiovascular mortality, non-cardiovascular mortality, and overall mortality. After including the C-factor and the calcification volumes of the five vessel beds simultaneously into a model, the C-factor was still associated with the three types of mortality (adjusted hazard ratio per standard deviation increase of the fourth-root-transformed C-factor (HR) > 1.52). In contrast, the associations of the separate vessel beds with mortality attenuated considerably (HR < 1.26) (Table 3, Model 3). The associations between the C-factor, the calcification volumes in the separate vessel beds and mortality did not change remarkably after excluding participants with prevalent cardiovascular disease (Additional file 1: Table S3).

**Table 3:** The C-factor, calcification in different vessel beds and risk all-cause and cause-specific mortality

	Cardiovascular mortality			Non-cardiovascular mortality			Overall mortality		
	n/N = 121/2384 HR per SD (CI)			n/N = 335/2384 HR per SD (CI)			n/N = 456/2384 HR per SD (CI)		
	Model 1	Model 2	Model 3	Model 1	Model 2	Model 3	Model 1	Model 2	Model 3
$\sqrt[4]{\text{C-factor}}$	1.94 (1.58; 2.38)	1.80 (1.46; 2.23)	1.61 (0.88; 2.96)	1.41 (1.25; 1.59)	1.40 (1.24; 1.59)	1.52 (1.06; 2.18)	1.53 (1.38; 1.70)	1.49 (1.34; 1.66)	1.56 (1.14; 2.12)
CAC*	1.87 (1.47; 2.38)	1.77 (1.38; 2.27)	1.26 (0.91; 1.75)	1.27 (1.12; 1.45)	1.27 (1.12; 1.45)	0.98 (0.82; 1.17)	1.40 (1.25; 1.56)	1.37 (1.22; 1.54)	1.04 (0.89; 1.22)
AAC*	1.67 (1.26; 2.21)	1.49 (1.13; 1.97)	0.96 (0.68; 1.35)	1.40 (1.21; 1.62)	1.37 (1.18; 1.59)	1.08 (0.88; 1.33)	1.46 (1.28; 1.66)	1.39 (1.22; 1.59)	1.04 (0.88; 1.24)
ECAC*	1.62 (1.30; 2.01)	1.49 (1.20; 1.85)	1.03 (0.76; 1.40)	1.27 (1.12; 1.43)	1.24 (1.10; 1.40)	0.97 (0.81; 1.15)	1.34 (1.21; 1.49)	1.30 (1.17; 1.44)	0.98 (0.85; 1.14)
ICAC*	1.65 (1.29; 2.11)	1.52 (1.19; 1.95)	0.94 (0.67; 1.31)	1.20 (1.06; 1.36)	1.18 (1.04; 1.34)	0.87 (0.73; 1.05)	1.29 (1.16; 1.45)	1.25 (1.12; 1.40)	0.88 (0.75; 1.03)
VBAC*	1.33 (1.18; 1.51)	1.28 (1.13; 1.46)	1.10 (0.94; 1.28)	1.14 (1.05; 1.25)	1.13 (1.03; 1.24)	1.02 (0.92; 1.13)	1.20 (1.12; 1.28)	1.17 (1.09; 1.26)	1.05 (0.96; 1.14)

AAC, aortic arch calcification; CAC, coronary artery calcification; CI, 95%-confidence interval; ECAC, extracranial carotid artery calcification; ICAC, intracranial carotid artery calcification; HR, hazard ratio; n, cases; N, persons at risk; SD, standard deviation; and VBAC, vertebrobasilar artery calcification

Model 1: Adjusted for age and sex

Model 2: Adjusted for age, sex, current smoking status, obesity, diabetes mellitus, hypertension, hypercholesterolemia and low high-density lipoprotein cholesterol

Model 3: Adjusted for age, sex, the C-factor and calcification in all vessel beds

*All transformed volumes,( $\sqrt[4]{C - factor}$ (after transformation to solely positive values)) in case of the C-factor and ln(calcification volume + 1 mm^3^) in case of calcification in the separate vessel beds

## Discussion

In this study, we showed that the C-factor captures the systemic burden of arterial calcification into a single measure. The C-factor explained consistently more than half of the variance between variables included in the analyses. Furthermore, the C-factor remained robust across different combinations of vessel beds used for its construction. The latter has practical advantages as well. The C-factor introduces the opportunity to make a reasonable estimate of systemic calcification with information on calcification volumes in a limited number, two or more, of vessel beds. Since some vessel beds are captured on the same CT-scan, the C-factor provides an alternative, with only a need for limited scan volume, to determine the systemic component of arterial calcification.

Previous studies reported the associations between arterial calcification and cardiovascular, non-cardiovascular, and overall mortality [18, 28]. Given that we believe with the C-factor we are able to capture systemic calcification burden, we explicitly linked it to the broad outcome of mortality and not to specific outcomes such as CHD or stroke. Our study showed that when systemic arterial calcification and calcification volumes in the separate vessel beds were put in the same model, the association of the C-factor with all three types of mortality was durable. In comparison, the associations of calcification in the separate vessel beds with non-cardiovascular and overall mortality weakened. This could be an indication that systemic arterial calcification represents an important link between arterial calcification and fatal diseases even beyond traditional cardiovascular diseases. Moreover, based on the consistent association between the C-factor and non-cardiovascular death, we believe that the C-factor may even reflect a person’s overall health status more accurately. Further research into the relationship between systemic calcification and fatal diseases is needed, but the C-factor can be used to capture the risk that systemic arterial calcification entails.

The main strengths of this study are its population-based study design, complete follow-up data on mortality, the large study population, and the relatively easily applicable statistical method to compose the C-factor. Moreover, we found that the C-factor did not differ between men and women, suggesting that the C-factor can be used in both men and women. However, our study had some limitations. First of all, we only included calcification in five vessel beds in this study. We did not have information on calcification in, for instance, the legs or the abdomen. Further research is needed to conclude if the C-factor remains robust when other vessel beds are included in the principal component analysis. Furthermore, by scanning five instead of one vessel bed, the participants were exposed to a higher amount of radiation [3]. However, the scans were performed between 2003 and 2006. The radiation exposure is lower when using newer generation scanners [29, 30]. Further research into the C-factor’s predictive value is needed to determine if the benefits of the C-factor outweigh the higher amount of radiation exposure. Secondly, the C-factor does not capture non-calcified plaque, since a non-calcified plaque will not influence the amount of arterial calcification. Still, as calcified arteriosclerosis is by far the most common subtype of arteriosclerosis, we believe it provides the best possible estimate of arteriosclerosis. Thirdly, we did not have information on serum biomarkers for calcification or the calcium score in all vessel beds in the participants. We believe it would be interesting to compare the performance of the C-factor with serum biomarkers and the calcium score in future research. Fourthly, our study population consisted mostly of white and older individuals. Earlier research showed differences in calcification among different ethnic populations [31, 32]. The C-factor needs to be investigated in other ethnic populations to determine if the systemic component of arterial calcification differs among ethnic populations. Besides that, we selected our study population based on the visit of participants to the research center. Hereby we might have missed a small proportion of the population who had an extremely high overall calcification score as they were too ill to attend. This could have led to a small underestimation of the true effect from the risk of an elevated C-factor on mortality. Finally, we measured the C-factor only cross-sectionally and have, therefore, no information on the trajectories of the C-factor. This paper is, most of all, a proof of principle. It is possible to capture calcification in multiple vessel beds in one summary measure. We believe future research with information on the C-factor on multiple time points is needed to get insights on how the calcification burden changes over time.

With the C-factor, we composed a single summary measure to determine the systemic component of arterial calcification on an individual level, which can be used in etiologic research and as a predictor in other studies. We believe that the C-factor can also be useful to monitor the effects of systemic medical treatment for cardiovascular risk reduction in trials or clinical practice as it captures systemic arterial calcification. However, further research on and validation of the C-factor is needed to confirm the C-factor as a systemic arterial calcification summary measure.

## Supplementary Information

**Additional file 1: Table S1.** Sensitivity analysis: creating a sex-specific C-factor. A multi-page table displaying the loadings of the different vessel beds included in the C-factors when composed based on the entire population, and when only the men or women were used to compose the C-factors. **Table S2.** Descriptive statistics of the different C-factors. A multi-page table describing the descriptive statistics of all 26 possible combinations of the vessel beds included in this study to compose the C-factors. **Table S3.** The C-factor, calcification in different vessel beds and risk all-cause and cause-specific mortality in participants without prevalent cardiovascular disease. A landscape table to describe the associations between cardiovascular, non-cardiovascular and overall mortality and the C-factor and calcification in the different vessel beds in participants without prevalent cardiovascular disease at the moment of the CT-scan.

## Data Availability

Requests to access the data set from qualified researchers trained in human subject confidentiality protocols may be sent to the Department of Epidemiology, Erasmus MC University Medical Center at f.vanrooij@erasmusmc.nl.

## Abbreviations

AAC: Aortic arch calcification
CAC: Coronary artery calcification
CT: Computed tomography
ECAC: Extracranial artery calcification
HR: Hazard ratio
ICAC: Intracranial carotid artery calcification
PCA: Principal component analysis without varimax rotation
SD: Standard deviation
VBAC: Vertebrobasilar artery calcification
