# Supplementary material for: C-factor: a summary measure for systemic arterial calcifications
Source: BMC Cardiovasc Disord. 2021 Jun 29;21:317. doi: 10.1186/s12872-021-02126-y (PMC8243490; doi:10.1186/s12872-021-02126-y)
Supplement: Supplementary file 1 — Additional file 1: Table S1. Sensitivity analysis: creating a sex-specific C-factor. A multi-page table displaying the loadings of the different vessel beds included in the C-factors when composed based on the entire population, and when only the men or women were used to compose the C-factors. Table S2. Descriptive statistics of the different C-factors. A multi-page table describing the descriptive statistics of all 26 possible combinations of the vessel beds included in this study to compose the C-factors. Table S3. The C-factor, calcification in different vessel beds and risk all-cause and cause-specific mortality in participants without prevalent cardiovascular disease. A landscape table to describe the associations between cardiovascular, non-cardiovascular and overall mortality and the C-factor and calcification in the different vessel beds in participants without prevalent cardiovascular disease at the moment of the CT-scan. [file 12872_2021_2126_MOESM1_ESM.docx]

# “ADDITIONAL FILE 1”

C-factor: a summary measure for systemic arterial calcifications

Short title: Systemic arterial calcification biomarker

Lieke M. Kuiper, MSc, MA^1^; M. Kamran Ikram, MD, PhD^1,3^, Maryam Kavousi, MD, PhD^1^; Meike W. Vernooij, MD, PhD^1,2^; M. Arfan Ikram, MD, PhD^1*^; Daniel Bos, MD, PhD^1,2*^

*Joint last authorship

Departments of ^1^Epidemiology, ^2^Radiology and Nuclear Medicine and ^3^Neurology, Erasmus MC, Rotterdam, the Netherlands

**Additional file 1: Table S1**

Sensitivity analysis: creating a sex-specific C-factor.

|  |  | Composed based on entire population | Composed based on men | Composed based on women |
| --- | --- | --- | --- | --- |
| Combination-specific C-factor based on | Vessel bed | Loading | Loading | Loading |
| CAC, AAC, ECAC, ICAC, VBAC | CAC | 0.447 | 0.432 | 0.479 |
|  | AAC | 0.474 | 0.489 | 0.464 |
|  | ECAC | 0.489 | 0.490 | 0.479 |
|  | ICAC | 0.496 | 0.488 | 0.511 |
|  | VBAC | 0.299 | 0.311 | 0.254 |
| CAC, AAC,  ECAC, ICAC | CAC | 0.469 | 0.458 | 0.492 |
|  | AAC | 0.503 | 0.520 | 0.483 |
|  | ECAC | 0.523 | 0.527 | 0.504 |
|  | ICAC | 0.503 | 0.492 | 0.520 |
| CAC, AAC, ECAC, VBAC | CAC | 0.517 | 0.501 | 0.546 |
|  | AAC | 0.558 | 0.569 | 0.560 |
|  | ECAC | 0.563 | 0.565 | 0.551 |
|  | VBAC | 0.321 | 0.324 | 0.292 |
| CAC, ECAC, ICAC, VBAC | CAC | 0.514 | 0.502 | 0.540 |
|  | ECAC | 0.524 | 0.521 | 0.520 |
|  | ICAC | 0.568 | 0.567 | 0.583 |
|  | VBAC | 0.372 | 0.393 | 0.314 |
| CAC, AAC, ICAC, VBAC | CAC | 0.514 | 0.496 | 0.554 |
|  | AAC | 0.511 | 0.520 | 0.507 |
|  | ICAC | 0.565 | 0.562 | 0.568 |
|  | VBAC | 0.394 | 0.410 | 0.337 |
| AAC, ECAC, ICAC, VBAC | AAC | 0.533 | 0.540 | 0.531 |
|  | ECAC | 0.543 | 0.538 | 0.556 |
|  | ICAC | 0.549 | 0.538 | 0.566 |
|  | VBAC | 0.347 | 0.360 | 0.298 |
| CAC, AAC, ECAC, | CAC | 0.538 | 0.525 | 0.560 |
|  | AAC | 0.590 | 0.600 | 0.587 |
|  | ECAC | 0.602 | 0.604 | 0.585 |
| CAC, AAC, ICAC | CAC | 0.569 | 0.561 | 0.589 |
|  | AAC | 0.571 | 0.583 | 0.549 |
|  | ICAC | 0.592 | 0.587 | 0.593 |
| CAC, AAC, VBAC | CAC | 0.625 | 0.616 | 0.647 |
|  | AAC | 0.618 | 0.626 | 0.625 |
|  | VBAC | 0.476 | 0.478 | 0.437 |
| CAC, ECAC, ICAC | CAC | 0.560 | 0.558 | 0.566 |
|  | ECAC | 0.582 | 0.588 | 0.562 |
|  | ICAC | 0.589 | 0.586 | 0.603 |
| CAC, ECAC, VBAC | CAC | 0.642 | 0.636 | 0.659 |
|  | ECAC | 0.624 | 0.626 | 0.622 |
|  | VBAC | 0.446 | 0.453 | 0.423 |
| CAC, ICAC, VBAC | CAC | 0.580 | 0.557 | 0.624 |
|  | ICAC | 0.636 | 0.638 | 0.644 |
|  | VBAC | 0.509 | 0.532 | 0.444 |
| AAC, ECAC, ICAC | AAC | 0.577 | 0.589 | 0.561 |
|  | ECAC | 0.595 | 0.595 | 0.594 |
|  | ICAC | 0.559 | 0.547 | 0.577 |
| AAC, ECAC VBAC | AAC | 0.657 | 0.658 | 0.667 |
|  | ECAC | 0.648 | 0.646 | 0.656 |
|  | VBAC | 0.386 | 0.388 | 0.354 |
| AAC, ICAC, VBAC | AAC | 0.579 | 0.573 | 0.603 |
|  | ICAC | 0.641 | 0.636 | 0.650 |
|  | VBAC | 0.504 | 0.517 | 0.463 |
| ECAC, ICAC, VBAC | ECAC | 0.588 | 0.573 | 0.621 |
|  | ICAC | 0.654 | 0.651 | 0.669 |
|  | VBAC | 0.475 | 0.499 | 0.409 |
| CAC, AAC | CAC | 0.707 | 0.707 | 0.707 |
|  | AAC | 0.707 | 0.707 | 0.707 |
| CAC, ECAC | CAC | 0.707 | 0.707 | 0.707 |
|  | ECAC | 0.707 | 0.707 | 0.707 |
| CAC, ICAC | CAC | 0.707 | 0.707 | 0.707 |
|  | ICAC | 0.707 | 0.707 | 0.707 |
| CAC, VBAC | CAC | 0.707 | 0.707 | 0.707 |
|  | VBAC | 0.707 | 0.707 | 0.707 |
| AAC, ECAC | AAC | 0.707 | 0.707 | 0.707 |
|  | ECAC | 0.707 | 0.707 | 0.707 |
| AAC, ICAC | AAC | 0.707 | 0.707 | 0.707 |
|  | ICAC | 0.707 | 0.707 | 0.707 |
| AAC, VBAC | AAC | 0.707 | 0.707 | 0.707 |
|  | VBAC | 0.707 | 0.707 | 0.707 |
| ECAC, ICAC | ECAC | 0.707 | 0.707 | 0.707 |
|  | ICAC | 0.707 | 0.707 | 0.707 |
| ECAC, VBAC | ECAC | 0.707 | 0.707 | 0.707 |
|  | VBAC | 0.707 | 0.707 | 0.707 |
| ICAC, VBAC | ICAC | 0.707 | 0.707 | 0.707 |
|  | VBAC | 0.707 | 0.707 | 0.707 |

AAC indicates aortic arch calcification; CAC, coronary artery calcification; ECAC, extracranial carotid artery calcification; ICAC, intracranial internal carotid arteries; and VBAC, vertebrobasilar arteries.

**Additional file 1: Table S2**

Descriptive statistics of the different C-factors.

| Combination-specific C-factor based on | Mean | Median | 25^th^ Percentile | 75^th^ Percentile | IQR | Min | Max |
| --- | --- | --- | --- | --- | --- | --- | --- |
| CAC, AAC, ECAC, ICAC, VBAC | 0.00 | -0.63 | -0.97 | 0.33 | 1.29 | -1.12 | 14.03 |
| CAC, AAC, ECAC, ICAC | 0.00 | -0.61 | -0.96 | 0.34 | 1.29 | -1.11 | 13.45 |
| CAC, AAC, ECAC, VBAC | 0.00 | -0.56 | -0.84 | 0.20 | 1.04 | -0.93 | 13.55 |
| CAC, ECAC, ICAC, VBAC | 0.00 | -0.57 | -0.85 | 0.23 | 1.07 | -0.94 | 14.02 |
| CAC, AAC, ICAC, VBAC | 0.00 | -0.57 | -0.85 | 0.26 | 1.12 | -0.98 | 14.43 |
| AAC, ECAC, ICAC, VBAC | 0.00 | -0.56 | -0.87 | 0.22 | 1.09 | -1.00 | 14.35 |
| CAC, AAC, ECAC | 0.00 | -0.53 | -0.82 | 0.21 | 1.03 | -0.92 | 13.31 |
| CAC, AAC, ICAC | 0.00 | -0.55 | -0.85 | 0.32 | 1.17 | -0.99 | 11.76 |
| CAC, AAC, VBAC | 0.00 | -0.48 | -0.70 | 0.15 | 0.85 | -0.77 | 13.20 |
| CAC, ECAC, ICAC | 0.00 | -0.55 | -0.84 | 0.25 | 1.09 | -0.94 | 12.68 |
| CAC, ECAC, VBAC | 0.00 | -0.51 | -0.68 | 0.13 | 0.81 | -0.72 | 12.42 |
| CAC, ICAC, VBAC | 0.00 | -0.50 | -0.71 | 0.11 | 0.83 | -0.78 | 14.74 |
| AAC, ECAC, ICAC | 0.00 | -0.54 | -0.86 | 0.25 | 1.11 | -0.99 | 12.28 |
| AAC, ECAC, VBAC | 0.00 | -0.49 | -0.72 | 0.13 | 0.85 | -0.79 | 14.05 |
| AAC, ICAC, VBAC | 0.00 | -0.48 | -0.74 | 0.17 | 0.91 | -0.84 | 15.50 |
| ECAC, ICAC, VBAC | 0.00 | -0.50 | -0.73 | 0.13 | 0.85 | -0.79 | 15.05 |
| CAC, AAC | 0.00 | -0.46 | -0.70 | 0.16 | 0.86 | -0.77 | 11.80 |
| CAC, ECAC | 0.00 | -0.49 | -0.68 | 0.14 | 0.81 | -0.71 | 12.98 |
| CAC, ICAC | 0.00 | -0.49 | -0.72 | 0.19 | 0.91 | -0.79 | 10.90 |
| CAC, VBAC | 0.00 | -0.40 | -0.48 | -0.04 | 0.44 | -0.48 | 16.84 |
| AAC, ECAC | 0.00 | -0.48 | -0.71 | 0.16 | 0.87 | -0.78 | 13.08 |
| AAC, ICAC | 0.00 | -0.48 | -0.75 | 0.24 | 0.99 | -0.86 | 9.38 |
| AAC, VBAC | 0.00 | -0.38 | -0.52 | 0.03 | 0.55 | -0.55 | 15.84 |
| ECAC, ICAC | 0.00 | -0.49 | -0.73 | 0.16 | 0.89 | -0.80 | 11.09 |
| ECAC, VBAC | 0.00 | -0.39 | -0.49 | 0.00 | 0.49 | -0.49 | 14.06 |
| ICAC, VBAC | 0.00 | -0.39 | -0.54 | 0.02 | 0.56 | -0.57 | 16.81 |

AAC indicates aortic arch calcification; CAC, coronary artery calcification; ECAC, extracranial carotid artery calcification; ICAC, intracranial carotid artery calcification; IQR, interquartile range; Max, the sample maximum; Min, the sample minimum; and VBAC, vertebrobasilar artery calcification.

**Additional file 1: Table S3**

The C-factor, calcification in different vessel beds and risk all-cause and cause-specific mortality in participants without prevalent cardiovascular disease.

|  | Cardiovascular mortality | | | Non-cardiovascular mortality | | | Overall mortality | | | |
| --- | --- | --- | --- | --- | --- | --- | --- | --- | --- | --- |
|  | n/N = 83/2135  HR per SD (CI) | | | n/N = 273/2135  HR per SD (CI) | | | n/N = 356/2135  HR per SD (CI) | | | |
|  | Model 1 | Model 2 | Model 3 | Model 1 | Model 2 | Model 3 | Model 1 | Model 2 | Model 3 |  |
| $\sqrt[4]{C-factor}$ | 1.70 (1.32;2.18) | 1.59 (1.23;2.05) | 1.54 (0.76;3.12) | 1.33 (1.17;1.52) | 1.31 (1.14;1.49) | 1.36 (0.92;2.00) | 1.40 (1.25;1.57) | 1.36 (1.21;1.53) | 1.39 (0.99;1.95) |  |
| CAC* | 1.59 (1.23;2.07) | 1.53 (1.18;2.00) | 1.21 (0.86;1.71) | 1.21 (1.05;1.38) | 1.19 (1.04;1.37) | 0.98 (0.82;1.18) | 1.28 (1.14;1.44) | 1.26 (1.11;1.42) | 1.03 (0.88;1.21) |  |
| AAC* | 1.53 (1.10;2.13) | 1.39 (1.01;1.93) | 0.99 (0.65;1.53) | 1.31 (1.12;1.54) | 1.28 (1.09;1.50) | 1.07 (0.86;1.33) | 1.35 (1.17;1.56) | 1.29 (1.12;1.49) | 1.05 (0.86;1.28) |  |
| ECAC* | 1.40 (1.10;1.79) | 1.33 (1.04;1.69) | 0.99 (0.70;1.39) | 1.24 (1.09;1.41) | 1.21 (1.06;1.38) | 1.02 (0.85;1.23) | 1.27 (1.14;1.43) | 1.23 (1.10;1.38) | 1.02 (0.86;1.20) |  |
| ICAC* | 1.38 (1.05;1.83) | 1.31 (0.99;1.73) | 0.90 (0.62;1.30) | 1.15 (1.01;1.32) | 1.14 (1.00;1.30) | 0.90 (0.74;1.09) | 1.20 (1.06;1.24) | 1.17 (1.04;1.32) | 0.89 (0.75;1.06) |  |
| VBAC* | 1.25 (1.07;2.05) | 1.22 (1.05;1.43) | 1.08 (0.90;1.29) | 1.11 (1.01;1.22) | 1.11 (1.01;1.22) | 1.02 (0.91;1.14) | 1.15 (1.06;1.24) | 1.14 (1.05;1.24) | 1.04 (0.95;1.14) |  |

AAC indicates aortic arch calcification; CAC, coronary artery calcification; CI, 95%-confidence interval; ECAC, extracranial carotid artery calcification; ICAC, HR, hazard ratio; intracranial carotid artery calcification; n, cases; N, persons at risk; SD, standard deviation; and VBAC, vertebrobasilar artery calcification.

Model 1: Adjusted for age and sex.

Model 2: Adjusted for age, sex, current smoking status, obesity, diabetes mellitus, hypertension, hypercholesterolemia and low high-density lipoprotein cholesterol.

Model 3: Adjusted for age, sex, the C-factor and calcification in all vessel beds.

* All transformed volumes, $\sqrt[4]{C-factor after translation to solely positive outcomes}$) in case of the C-factor and ln(calcification volume + 1 mm^3^) in case of calcification in the separate vessel beds.
